# Ultrasound-mediated nanobubble destruction (UMND) facilitates the delivery of A10-3.2 aptamer targeted and siRNA-loaded cationic nanobubbles for therapy of prostate cancer

**DOI:** 10.1080/10717544.2017.1422300

**Published:** 2018-01-09

**Authors:** Meng Wu, Hongyun Zhao, Liang Guo, Yiru Wang, Jiao Song, Xueli Zhao, Chongyan Li, Lan Hao, Dong Wang, Jie Tang

**Affiliations:** a Department of Ultrasound, Chinese PLA General Hospital, Beijing, China;; b School of Medicine, Nankai University, Tianjin, China;; c Department of Gastroenterology, The Second Affiliated Hospital of Chongqing Medical University & Chongqing Key Laboratory of Ultrasound Molecular Imaging, Chongqing, China;; d Department of Obstetrics and Gynecology, The Second Affiliated Hospital of Chongqing Medical University, Chongqing, China;; e Ultrasound Department, Xijing Hospital, Fourth Military Medical University, Xi’an, China;; f State Key Laboratory of Ultrasound Engineering in Medicine Co-Founded by Chongqing and the Ministry of Science and Technology, Chongqing Medical University, Chongqing, China;; g Chongqing Key Laboratory of Ultrasound Molecular Imaging, The Second Affiliated Hospital of Chongqing Medical University, Chongqing, China;; h Department of Ultrasound, The First Affiliated Hospital of Chongqing Medical University, Chongqing, China

**Keywords:** Ultrasound-mediated nanobubble destruction, gene silencing, aptamer, tumor targeting, prostate cancer

## Abstract

The Forkhead box M1 (FoxM1) transcription factor is an important anti-tumor target. A novel targeted ultrasound (US)-sensitive nanobubble that is likely to make use of the physical energy of US exposure for the improvement of delivery efficacy to target tumors and specifically silence FoxM1 expression appears as among the most potential nanocarriers in respect of drug delivery. In this study, we synthesized a promising anti-tumor targeted FoxM1 siRNA-loaded cationic nanobubbles (CNBs) conjugated with an A10-3.2 aptamer (siFoxM1-Apt-CNBs), which demonstrate high specificity when binding to prostate-specific membrane antigen (PSMA) positive LNCaP cells. Uniform nanoscaled siFoxM1-Apt-CNBs were developed using a thin-film hydration sonication, carbodiimide chemistry approaches, and electrostatic adsorption methods. Fluorescence imaging as well as flow cytometry evidenced the fact that the siFoxM1-Apt-CNBs were productively developed and that they specifically bound to PSMA-positive LNCaP cells. siFoxM1-Apt-CNBs combined with ultrasound-mediated nanobubble destruction (UMND) significantly improved transfection efficiency, cell apoptosis, and cell cycle arrest *in vitro* while reducing FoxM1 expression. *In vivo* xenografts tumors in nude-mouse model results showed that siFoxM1-Apt-CNBs combined with UMND led to significant inhibition of tumor growth and prolonged the survival of the mice, with low toxicity, an obvious reduction in FoxM1 expression, and a higher apoptosis index. Our study suggests that siFoxM1-Apt-CNBs combined with UMND might be a promising targeted gene delivery strategy for therapy of prostate cancer.

## Introduction

Prostate cancer is termed as the frequently found malignant kind of cancer occurring in the male reproductive system, and these tumors frequently recurrence after surgery, radiotherapy and chemotherapy. RNA interference (RNAi) can specifically reduce high oncogene expression in prostate cancer cells and serve as a powerful tool in respect of gene treatment (Oh & Park, 2009). Nevertheless, this method is not capable of being utilized now in medical treatments (He et al., 2017). Productive gene therapy needs elevated high gene transfection efficacy as well as expression (Zhou et al., 2015). Viral vectors, indicated by adenoviruses as well as lentiviruses, possess extensively elevated transduction efficacy; nonetheless, their latent infection, together with immunogenicity threats has not been productively resolved, thus far. Despite the fact that liposome transfection performs a quintessential function in *in vitro* cell research works, yet its transfection efficacy is quite low, together with high cost. Accordingly, the application is only experimental.

For the purpose of overcoming these disadvantages, other physical as well as chemical methodologies have been suggested for enhancing the gene transfection efficacy. In particular, ultrasound-mediated microbubble destruction (UMMD) technology is able to particularly destruct ultrasound contrast agents (UCAs) in US-irradiated locations, in addition to playing a substantial function in the gene treatment.

Nevertheless, the key issue associated with this technology suggests that its low transfection efficiency constrains its application; accordingly, majority of the scholars have emphasized methods of improving the efficiency of gene transfection. Nonetheless, former research in this area has primarily focused on microscale UCAs; in addition, few research is available that has investigated the currently established nanoscale UCAs. Nanoscale UCAs are defined as the contrast agents having a specific diameter of below 700 nm that can be utilized for contrast-enhanced US (CEUS) imaging. In comparison with the microscale UCAs, nanoscale UCAs possess attributes of robust penetration capacity, together with extraordinary stability; more extensive distribution of nanoscale UCAs can be made in tumor tissues with the use of improved enhanced permeability and retention (EPR) effect impacts: huge vascular endothelial spaces, imperfect basement membranes, as well as inadequate lymphatic drainage. Accordingly, they have been extensively researched, particularly in solid-tumor applications (Wang et al., 2010; Xing et al., 2010). Nanoscale UCAs have exogenous cavitation nuclei and lower the ultrasound power threshold that is required for sonoporation (Greenleaf et al., 1998; Miller et al., 2002), in addition to being capable of serving as vectors. Ordinary NBs are based on film-forming materials that are primarily lipids and proteins, and the surface potentials of these NBs are mostly neutral or weakly negative; these types of NBs are called neutral NBs (NNBs) (Nomikou et al., 2012). When DNA/RNA is loaded onto a NNB by electrostatic adsorption, the DNA/RNA is also negatively charged, which is not conducive to the binding of the DNA/RNA to the NNB. Moreover, the loading gene capacity of the NNB is low, and the negative charge of the DNA/RNA is not conducive to the contact that exists between the NNB and the cells with negative charge. Thus, the transfection efficiency is not high. As a useful gene vector, NBs have an ideal surface with a positive charge, which can improve the combination of the NB with negatively charged nucleic acids or cells. Cationic cholesterol has attracted increasing attention in recent years, having an extremely positive charge, together with a robust potential of combining with negatively charged DNA or RNA and clearly enhance gene transfer. There have been reports that cationic lipids possess the cell toxicity, although a number of scholars do not agree that cationic bubbles are toxic to cells (Nomikou & McHale, 2010; Wang et al., 2012b; Sun et al., 2013).

In ultrasound-mediated nanobubble destruction (UMND) combined with CNBs for gene therapy, the use of CNB can be made not just as a carrier for the improvement of the gene that carries potential but also for protecting the gene from DNase degradation in the serum. Due to the positively charged facades of CNB, the delivered gene is increasingly concentrated at the treatment locations (Wang et al., 2012b). There is one other methodology that can increase in the local gene concentration for the production of nanobubbles targeting aggregations. Therefore, the development of novel tumor-targeting agent has become more and more urgent. Nanobubbles have been linked to a particular targeting agent binding to disease-related molecular markers expressed in prostate cancer (Fan et al., 2016). We made a selection of an A10-3.2 aptamer that has just 39 nucleotides as the targeting agent that probably creates a bond of the targeted proteins with the use of the best specificity as well as resemblance, accordingly, modulates the role performed by the target protein (Faulhammer et al., 2004; Huang et al., 2007; Jeong et al., 2009). Aptamers, using the steady structures, together with low immunogenicity, are capable for chemical synthesis and stabilization (Fan et al., 2016). A10-3.2 aptamers conjugated to CNBs boost targeting, leading to more effective therapeutics and more discerning diagnostics. CNB conjugates with the A10-3.2 aptamer have been used to target prostate-specific membrane antigen (PSMA) (Farokhzad et al., 2004). Upregulation of this transmembrane protein occurs in metastatic prostate cancer, as well as androgen-independent prostate cancer (Lupold et al., 2002; Ristau et al., 2014). A10-3.2 aptamers functionalized onto CNBs perform a quintessential function to target these nanobubbles to a particular subset of prostate cancer cells (Wang et al., 2012a). Importantly, as revealed by the latest research works, Forkhead box M1 (FoxM1), a gene involved in proliferation, is overexpressed in prostate tumors (Wang et al., 2014). Inhibition of the FoxM1 protein not only inhibits proliferation but also promotes apoptosis. Therefore, FoxM1 has emerged as a fresh target of the gene treatment in prostate cancer (Kalin et al., 2006; Wang et al., 2007; Green et al., 2011; Kalin et al., 2011; Uddin et al., 2011; Liu et al., 2012).

In this research, we developed a multifunctional nanocarrier gene delivery system composed of (1) FoxM1siRNA regarding localized treatment, (2) CNBs regarding gene nanocarriers, (3) tumor-specific aptamers regarding cell targeting, and (4) ultrasound-mediated CNBs destruction to released siRNA. We evaluated the ability of the Apt-CNBs to carry FoxM1 siRNA, the US sensitivity, and the protective effect of the approach on the FoxM1 siRNA. Using *in vitro*, together with *in vivo* experiments, we evaluated the therapeutic impact of the siFoxM1-Apt-CNBs combined with UMND transfection therapy and the expression of FoxM1 and E-cadherin in LNCaP cells and xenografts tumor in nude mice. This evaluation brings forth a research basis supporting the hypothesis that siFoxM1-Apt-CNBs combined with UMND represent a promising targeted gene delivery strategy for treating prostate cancer.

## Materials and methods

### Materials

1,2-Dipalmitoyl-sn-glycero-3-phosphocholine (DPPC; MW, 734.039) and 3β-[N-(N′,N′-dimethylaminoethane)-carbamoyl]cholesterol hydrochloride (DC-Chol, MW, 537.260) were purchased from Avanti Polar Lipids Inc. (Alabaster, AL), and carboxyl-modified 1,2-distearoyl-sn-glycero-3-phosphoethanolamine was purchased from DSPE-PEG2000-COOH; MW, 2771, NANOCS (Boston, MA).

1,1′-Dioctadecyl-3,3,3′,3′-tetramethylindocarbocyanine perchlorate (DiI, MW, 933.88), 2-(4-amidinophenyl)-6-indolecarbamidine dihydrochloride (DAPI, MW, 350.25), and 3,3′-dioctadecyloxacarbocyanine perchlorate (DiO, MW, 881.72) were purchased from Beyotime Biotechnology (Shanghai, China).

Octafluoropropane (C_3_F_8_) gas was purchased from the Research Institute of Physical and Chemical Engineering of Nuclear Industry (Tianjin, China). Anti-FoxM1 and anti-E-cadherin antibodies were purchased from Abcam (Cambridge, UK). siRNA duplexes designed to target FoxM1 gene were purchased from Genepharma Co., Ltd. (Shanghai, China). These duplexes were 21-nt long double stranded RNA oligos, with dTdT 3′-end overhangs, and the sense and antisense sequences (5′ to 3′) were GUGUCUCGGAAAUGCUUGUTT and ACAAGCAUUUCCGAGACACTT, respectively.

A10-3.2 aptamer (5-GGGAGGACGAUGCGGAUCAGCCAUGUUUACGUCACUCCU-spacer-NH2-3′ with 2′-fluoro pyrimidines) was purchased from RiboBio Co., Ltd. (Guangzhou, China).

### Preparation of NBs

Fixed ratios of DPPC, DSPE-PEG2000-COOH, and DC-cholesterol were established by dissolving these components in 4 mL of chloroform. Then, 4 μL of DiI (1.0 mg/mL, Beyotime, Shanghai, China) was added to the solution, and the solution was transferred to a round-bottom flask and placed in a rotary vacuum evaporator at 50 °C for one hour under low pressure to remove the organic solvent and form a uniform lipid film. Then, 0.5 mL of hydration liquid consisting of glycerol and phosphate-buffered saline (PBS) (v/v = 1:9) was added to form a lipid suspension and then dispensed into a 1.5-mL tube with a sealed rubber cap. Then, the air in the tube was replaced with perfluoropropane gas using a 50-mL syringe equipped with a long fine needle. The tube was placed in a water bath at 42 °C for 30 min. Then, the suspension was vibrated for 90 seconds using a mechanical vibrator to produce the Dil-labeled-CNBs and sealed at 4 °C until further use (light was avoided during the preparation process).

Meanwhile, DPPC, DSPE-PEG2000 and DC-cholesterol were used to prepare the cationic nanobubbles (CNBs) (no Dil); DPPC and DSPE-PEG2000 were used to prepare the neutral nanobubbles (NNBs) (Dil- and no Dil-labeled-NNBs), as described above.

### Synthesis of Apt-CNBs

After the synthesis of the CNBs containing the DSPE-PEG2000-COOH lipid molecule, 0.4 M EDC and 0.1M NHS were inserted at the ratio of 1:1 (v/v), and the CNBs were incubated for 30 min on a shaker. Thereafter, an amine-modified A10-3.2 aptamer at a 10 mM concentration was inserted, followed by performance of the covalently coupling reaction at room temperature for a time period of 30 minutes. Subsequent to the accomplishment of the covalently coupling reaction, targeted CNBs (Apt-CNBs) were attained with the use of the centrifugal flotation methodology.

### Characterization of Apt-CNBs

One milliliter of the primary suspension of Apt-CNBs was diluted into 14 mL of PBS. A drop of diluted Dil-labeled-Apt-CNBs was observed using an laser confocal scanning microscopy (LCSM, A1 + R; Nikon Corporation, Tokyo, Japan). Simultaneously, a drop of diluted Apt-CNBs was used on electron microscopy (TEM, JEM-2100, JEOL, Japan) to observe the morphology of the Apt-CNBs.

The average size, zeta potential, and polydispersity index (PDI) of the three NBs were assessed using a dynamic light scattering (DLC) apparatus (ZS90; Malvern Instruments, Malvern, UK), and the concentrations of the three Dil-labeled-NBs were measured using a hemocytometer and optical microscope. The experiment was repeated at least three times.

### Stability of binding between the CNBs and A10-3.2 aptamer

Construction of the FAM-labeled A10-3.2 aptamer as well as DiI-labeled CNBs was performed using the methodology described above. Observation of the link between the FAM-labeled A10-3.2 aptamer and DiI-labeled CNBs was made with the use of LCSM (A1 + R; Nikon Corporation, Tokyo, Japan), followed by the measurement of the binding efficiencies with the use of flow cytometry (FCM; FACSVantage; BD, Franklin Lakes, NJ).

### Quantitative analysis of siFoxM1-loaded NBs

FoxM1 siRNA was divided into 10-μg, 20-μg, 30-μg, and 40-μg groups. The FoxM1 siRNA was added to 4 × 10^8^/mL Apt-CNBs, CNBs, or NNBs and fully mixed. The siRNA was then incubated at 4 °C for 30 min followed by 400 rpm/min centrifugation for 3 min at 4 °C and standing for 30 min. The upper suspension comprised the NBs that were combined with the FoxM1 siRNA, and the lower layer comprised the free FoxM1 siRNA. The concentration of the free FoxM1 siRNA in the lower suspension was measured using a nucleic acid protein detector (NanoDrop2000, Thermo Scientific, Wilmington, DE), and the amount of free FoxM1 siRNA was calculated. Each group was assessed three times, and the amount of gene loaded was calculated as (total siRNA input – free siRNA)/the number of NBs. The experiment was repeated three times.

### RNase protection assay

RNase ONE^TM^ ribonuclease (Promega, Madison, WI) was used to measure the ability of Apt-CNBs to prevent the enzymatic degradation of siRNA. Apt-CNBs and FoxM1 siRNA preparation methods were performed according to the best combination of the above procedures. The Apt-CNBs and FoxM1 siRNA complexes were resuspended in 1 mL of reaction buffer (no ribonuclease was present in the buffer). Four groups were established: FoxM1 siRNA-loaded Apt-CNBs with RNase (siFoxM1-Apt-CNBs RNase (+)), FoxM1 siRNA-loaded Apt-CNBs without RNase (siFoxM1-Apt-CNBs RNase (–)), naked FoxM1 siRNA with RNase (naked siFoxM1 RNase (+)), and naked FoxM1 siRNA without RNase (naked siFoxM1 RNase (–)). For the siFoxM1-Apt-CNBs RNase (+) and naked siFoxM1 siRNA RNase (+) groups, 0.1 units of ribonuclease were added to the buffer. A small amount of sample from each group was obtained and added to a 2% gel (W/V). Then, the structural integrity of the FoxM1 siRNA was verified in a TAE buffer after electrophoresis at 100 mV for 30 min. The naked siFoxM1 RNase (–) group was used as the control.

### Cell culture and mouse xenograft tumor models

LNCaP cells (human prostate cancer cells line) were from American Type Culture Collection (ATCC, Manassas, VA) and PC3 cells (human prostate cancer cells line) were obtained from Department of Urology, The First Affiliated Hospital of Chongqing Medical University (Chongqing, China). Cells were cultured using DMEM F12 culture medium containing 10% heat-inactivated fetal bovine serum at a 37 °C and 5% CO_2_ incubator. BALB/c nude mice (male, four to six weeks aged, ∼20 g) were provided from the Laboratory Animal Centre of Chongqing Medical University. Tumor cells were implanted into BALB/c nude mice for the growth into tumor xenografts. Resuspension of the cells (1 × 10^7^/mL) in 0.2 mL of PBS was done, followed by subcutaneous injection into the right hip. Twenty-one days after inoculation with the tumor cells, xenograft growth was successful (150 mm^3^), and experiments were performed according to the International Guiding Principles. Approval for all of the experimental and nude mouse treatments was received from the Animal Ethics Committee of Chongqing Medical University.

### *In vitro* evaluation of the target binding

A separate culture of the PSMA-positive LNCaP as well as PSMA-negative PC3 cells was grown in a six-well plate with coverslip at a concentration of 1 × 10^5^ cells/mL in all of the wells. Subsequent to 24 hours of culture, washing of the cells was done thrice with the use of PBS. Additionally, division of the cells were done into five key groups: group I, DiI-labeled siFoxM1-Apt-CNBs + LNCaP cells; group II, DiI-labeled siFoxM1-CNBs + LNCaP cells; group III, DiI-labeled siFoxM1-NNBs + LNCaP cells; group IV (the inhibition/competitive group), DiI-labeled siFoxM1-Apt-NNBs + A10-3.2 aptamer + LNCaP cells (A10-3.2 aptamer excess group); and group V, DiI-labeled siFoxM1-Apt-NNBs + PC3 cells. Following incubation for 30 min at room temperature, washing of the cells contained in the five groups was done thrice with the use of PBS, which was fixed in 4% paraformaldehyde for a period of 15 minutes, followed by staining using both DAPI and DiO in respect of visualization of the nuclei, as well as membrane, correspondingly. Imaging of the stained coverslips was performed with the use of LCSM. DiI, DiO, followed by showing red, green, and blue fluorescences by DAPI, correspondingly. Determination of the binding efficiencies between cells and siFoxM1-NBs was measured with the use of FCM.

### Detection of low-frequency US sensitivity *in vitro*

A 2% (w/v) agarose gel was prepared in order to be utilized in the *in vitro* ultrasonography gel model, and 500 μL of the siFoxM1-Apt-CNBs was diluted four-fold with PBS and added to the gel model. The siFoxM1-Apt-CNBs CEUS imaging effects were observed *in vitro* with the following equipment and parameters: the Esaote MyLab 90 ultrasonic diagnostic system, an L5-12 probe, MI 0.10, 88% gain, CnTI contrast mode, 1.5-cm depth, and US focusing on the siFoxM1-Apt-CNBs interface. Additionally, the procedure involved custom-developed low-frequency US (Chongqing Key Laboratory of Ultrasound Molecular Imaging, Chongqing Medical University, Chongqing, China) with destruction to siFoxM1-Apt-CNBs for 1 min (1 MHz, 1.0 W/cm^2^, duty cycle 50%). Contrast-enhanced US images were recorded before and after destruction with MyLab 90, using the DFY ultrasonic image quantitative analysis software (Chongqing Key Laboratory of Ultrasound Molecular Imaging, Chongqing Medical University, Chongqing, China). This process was repeated for the siFoxM1-CNBs, siFoxM1-NNBs, and SonoVue (the use of commercially found SonoVue MBs was made as controls) (Bracco Corporation, Milano, Italy) groups as above. Greyscale intensity changes were analyzed before and after destruction.

### Cell transfection

Approximately, 5 × 10^4^ cells/well LNCaP cells were seeded in 12-well plates. Incubation of the plates was carried out at a temperature of 37 °C for a period of 24 hours, and the siFoxM1-NBs were prepared using the method described above. Two hundred microliters of siFoxM1-NBs was added to each well, and the volume in each well was brought up to 1 mL using complete growth medium and then incubated for 10 minutes at room temperature. A 1-cm-thick medical US coupling agent was applied between the low-frequency US probe and the bottom of the 12-well plates. After the US probe was fixed, the US exposure began. The following low-frequency US parameters were used: 1 MHz, 1.0 w/cm^2^, a 50% duty cycle, as well as a 1-min exposure. After the US exposure, incubation of the cells was performed at a temperature of 37 °C for a period of 48 hours.

### Transfection efficiency measurements

Seeding of LNCaP cells was carried out in a 12-well plate at a concentration of 5 × 10^4^ cells/well and transfected with four groups: Cy3-labeled FoxM1 Negative Control (NC)-siRNA-Apt-CNBs (siNC-Apt-CNBs), Cy3-labeled FoxM1 NC-siRNA-CNBs (siNC-CNBs), Cy3-labeled FoxM1 NC-siRNA-NNBs (siNC-NNBs), and PBS (control). After transfection for 24 hours, the four groups of cells were collected separately, and after centrifugation, the cells were resuspended 500-μL PBS and were performed for measuring the siNC-NBs combined with UMND gene transfer efficiency with the help of FCM. Each group of cells was tested three times.

### Intracellular distribution

The distribution of the Cy3-FoxM1 NC-siRNA in the cells was examined by CLSM. LNCaP cells were grown in petri dishes at 1 × 10^5^/well at 37 °C overnight. After the six-hours Cy3-FoxM1 NC-siRNA transfection for the flow cytometric analysis of the transfection method, washing of the cells was done thrice using PBS. DAPI and DiO were used to stain the nuclei and cell membranes for 15 min to visualize the cellular localization of the Cy3-FoxM1 NC-siRNA. Cells were observed by CLSM. Cy3, DAPI, and DiO were excited at 514, 358, and 484 nm, respectively. The emission wavelengths of Cy3, DAPI, and DiO were 570, 461, and 501 nm, respectively.

### Cytotoxicity assays

Cell Counting Kit-8 (CCK8, Beyotime, Shanghai, China) was used to evaluate cytotoxicity *in vitro*. Seeding of LNCaP cells was carried out in a 96-well plate at a concentration of 5 × 10^3^ cells/well at 37 °C overnight. Additionally, division of the cells was done into two groups: group I, FoxM1 NC-siRNA-Apt-CNBs (siNC-Apt-CNBs), siNC-CNBs, siNC-NNBs, and PBS (Control) (at siNC-NBs volume 200 µL/well) incubated with LNCaP cells for 48 hours; group II, siNC-Apt-CNBs + US, siNC-CNBs + US, siNC-NNBs + US, and PBS (Control) (at siNC-NBs volume 200 µL/well, the transfection method is described above) incubated with LNCaP cells for 48 hours. Following washing of the cells contained in the two groups was done thrice with the use of PBS, 110 μL of new medium that contained 10 µL of CCK-8 were added to each well. Then, 96-well plates were incubated for one hour at 37 °C, measurement of the absorbance at 450 nm was carried out with the use of a microplate reader. Three repetitions of the experiment were performed.

### Cell apoptosis assay *in vitro*

A separate culture of the PSMA-positive LNCaP as well as PSMA-negative PC3 cells was carried out in a six-well plate at a concentration of 1 × 10^5^ cells/well regarding incubation overnight. The LNCaP and PC3 cells were divided into four groups, respectively: siFoxM1-Apt-CNBs, siFoxM1-CNBs, siFoxM1-NNBs, PBS (control) groups. The LNCaP and PC3 cells were transfected with FoxM1 siRNA as previously described. After the LNCaP and PC3 cells were transfected for 48 hours, the cells from the different groups were digested with 0.25% trypsin, and cell suspensions were collected. Following centrifugation at a rate of 1000 rpm for a period of five minutes, the supernatants were removed. The cells were resuspended in 50 μL of binding buffer. Five microliters of V-fluorescein isothiocyanate (FITC) (BestBio, Shanghai, China) as well as 5 μL of propidium iodide (PI) (BestBio, Shanghai, China) were added to each tube. The samples were then mixed, and the reactions proceeded in the dark for 15 min. Four hundred and fifty microliters of the binding buffer was mixed into the samples after the reactions were complete, and the samples were analyzed by FCM.

### Cell cycle *in vitro*

A separate culture of the PSMA-positive LNCaP as well as PSMA-negative PC3 cells was carried out into six-well plates at a concentration of 1 × 10^5^ cells/well regarding incubation overnight. Division of the LNCaP and PC3 cells was performed into four groups, respectively: siFoxM1-Apt-CNBs, siFoxM1-CNBs, siFoxM1-NNBs, and PBS (control) groups. The transfection method is described previously. After a 48-h incubation period, collection of the LNCaP and PC3 cells was done, followed by washing them thrice with precooled PBS. After adding 70% ethanol precooled at 4 °C overnight, cell cycle analysis was performed using FCM.

### Cell viability assay *in vitro*

CCK-8 was used to evaluate LNCaP cell viability after transfection. Seeding of the cells was performed into 96-well plates at a concentration of 5 × 10^3^ cells/well, followed by incubation at a temperature of 37 °C overnight. The grouping and transfection methods were the same as those stated earlier in terms of the cell apoptosis analysis. Subsequent to 48 hours of incubation, replacement of the medium was made with 110 μL of new medium that contained 10 µL of CCK-8. Subsequent to one hour of incubation, measurement of the absorbance at 450 nm was carried out with the use of a microplate reader. Three repetitions of the experiment were performed.

### Real-time polymerase chain reaction (RT-PCR)

Following 48 hours of transfection, extraction of total RNA from cells was done with the use of a TRIzol Reagent Kit (Invitrogen, Life Technologies, Rockville, MD). Reverse transcription of RNA to cDNA was carried out according to the Advantage^®^ RT-for-Kit instructions. The RT-PCR was performed under the following condition: 42°C for 60 min, followed by 70°C for 15 min and 16°C for 5 min. The prepared cDNA was then amplified by PCR using the following program: 3 min at 95°C, followed by 95 °C for 5 s, 56°C for 10 s, and 72°C for 25 s, and 39 cycles at 65°C for 5 s, then end with 95°C for 50 s. The primer sequences have been presented in supplementary Table S1.

### Western blot analysis

After transfection for 48 hours, extraction of total protein from the cells was carried out, and the protein concentrations were measured using a BCA Enhanced Protein Assay Kit (BCA, Beyotime, Shanghai, China). Segregation of the protein samples (10 µg) was performed by sodium dodecyl sulfate polyacrylamide gel electrophoresis (SDS-PAGE) on 12% (w/v) polyacrylamide gels, followed by the electrotransfer to polyvinylidene difluoride (PVDF) membranes (Millipore, Bedford, MA). Subsequent to that, incubation of the PVDF membranes was carried out using blocking buffer (5% skim milk) at RT overnight. Thereafter, incubation of the membranes was performed using blocking buffer that contained 1:1000 rabbit anti-FoxM1 antibody and 1:50 mouse anti-E-cadherin antibody at 4 °C of temperature overnight. Washing of the PVDF membranes was done thrice for five minutes using PBST, followed by incubation using horseradish-peroxidase (HRP)-conjugated goat anti-rabbit IgG (1:10,000) or goat anti-mouse IgG (1:10,000) in blocking buffer at room temperature for a period of one hours to visualize the signal. The protein expression levels were normalized to that of GAPDH, and the band intensities were measured using a chemiluminescence system (Tanon-5200, Shanghai, China).

### *In vivo* fluorescence imaging in xenograft tumors

Injection of DiR-labeled siFoxM1-Apt-CNBs, siFoxM1-CNBs, and siFoxM1-NNBs 200 µL was made into LNCaP tumor-bearing nude mice through the tail vein. Assessments of the variations in the fluorescence intensity in xenograft tumors subjected to the time gaps of 0, 5, 10, 15 as well as 30 minutes, were observed *in vivo* with the use of a tiny animal living fluorescence imaging apparatus (IVIS Lumina Series III; PerkinElmer Inc., Waltham, MA). The euthanasia of nude mice was carried out, followed by the removal of liver, spleen, kidney, heart, lung organ tissues and observed fluorescence intensity with the use of a tiny animal living fluorescence imaging apparatus. Injection of DiI-labeled siFoxM1-Apt-CNBs, siFoxM1-CNBs as well as siFoxM1-NNBs 200 µL was also administered into LNCaP tumor-bearing nude mice by the means of the tail vein. The euthanasia of nude mice was carried out, followed by the removal of tumor tissue, liver, spleen and transferred to the pathology department for cryosectioning. Staining of the every piece of the organ tissues was carried out with the use of DAPI for visualizing the cell nucleus. Thereafter, triplicate washing of the specimens was carried out using the normal saline following incubation for a period of five minutes at room temperature. Treatment was given to the organ tissues using antifade mounting medium, followed by observation of the fluorescence with the help of LCSM.

### Therapeutic effect on mouse xenograft tumor *in vivo*

LNCaP tumor-bearing nude mice were randomly divided into four groups: siFoxM1-Apt-CNBs, siFoxM1-CNBs, siFoxM1-NNBs, and PBS (control) groups (*n* = 6). A 30-µg suspension of FoxM1 siRNA with 4 × 10^8^/mL of Apt-CNBs was injected via the tail vein (500 µL, injection time less than 10 seconds) to initiate gene transfection (1 MHz, 2 W/cm^2^, as well as 50% DC, three minutes, 1 second on/1 second off). Treatment was performed every three days, for an aggregate of seven treatments. Every three days, Vernier caliper measurements of the tumor sizes were performed once, and tumor growth was calculated using the following formula: *V* = 0.5×*a*×*b*^2^, where ‘*V*’ is an indication of the tumor volume (mm^3^), ‘*a*’ denotes the long diameter (mm) of the tumor, whereas ‘*b*’ represents the short diameter (mm) of the tumor. Transfections were performed by low-frequency US. After seven treatments, the tumor inhibition rate (TIR) was also calculated using the formula TIR = (1 – *V*1/*V*2) × 100%, where ‘*V*1’ is the average tumor volume in the experimented group (mm^3^) and ‘*V*2’ is the representation of the average volume found in the control group (mm^3^). Subsequent to the treatment for seven times, the nude mouse was killed , followed by collection of serum for the measurement of the albumin (ALB), alanine aminotransferase (ALT), aspartate aminotransferase (AST), blood urea nitrogen (BUN), as well as creatinine (CREA). Measurements of the cumulative survival time as well as body weight were taken in another group of xenografted tumors in nude mice every day after seven times gene therapy (*n* = 6).

### Immunohistochemical and TUNEL, PCNA analyses

Full tumor removal was performed after the seven treatments, and the animals were sacrificed thereafter. The tumor tissues were fixed in 4% paraformaldehyde, dehydrated via paraffin embedding, and biopsied. Immunohistochemistry was performed to evaluate FoxM1 and E-cadherin expression in the tumor tissues, and TdT-mediated dUTP nick-End Labelling (TUNEL) and proliferating cell nuclear antigen (PCNA) assays were carried out as well to assess apoptosis and proliferation in the tumor tissues.

FoxM1 and E-cadherin expression and the TUNEL and PCNA levels were detected through microscopy (BX51TF, Olympus, Tokyo, Japan). The proliferating and apoptotic positive cell count was semi-quantitatively attained through the count of the quantity of the cells that had positive stains from a minimum of five indiscriminately chosen high-power areas with the help of unaware observers. The proliferating index (PI), together with apoptotic index (AI) that were stated as the ratio of positively stained tumor cells to all cells were figured out from the minimum of five erratically chosen high-power areas.

### Statistical analyses

Statistical analyses were performed using one-way analysis of variance (ANOVA) (SPSS Software, version 20.0; IBM Inc., Armonk, NY, USA). All data are reported as the mean ± SD. A *p* value < .05 indicated a significant difference. All statistical tests were two-tailed.

## Results

### Evaluation of the binding between aptamer and NBs

**Figure 1.**
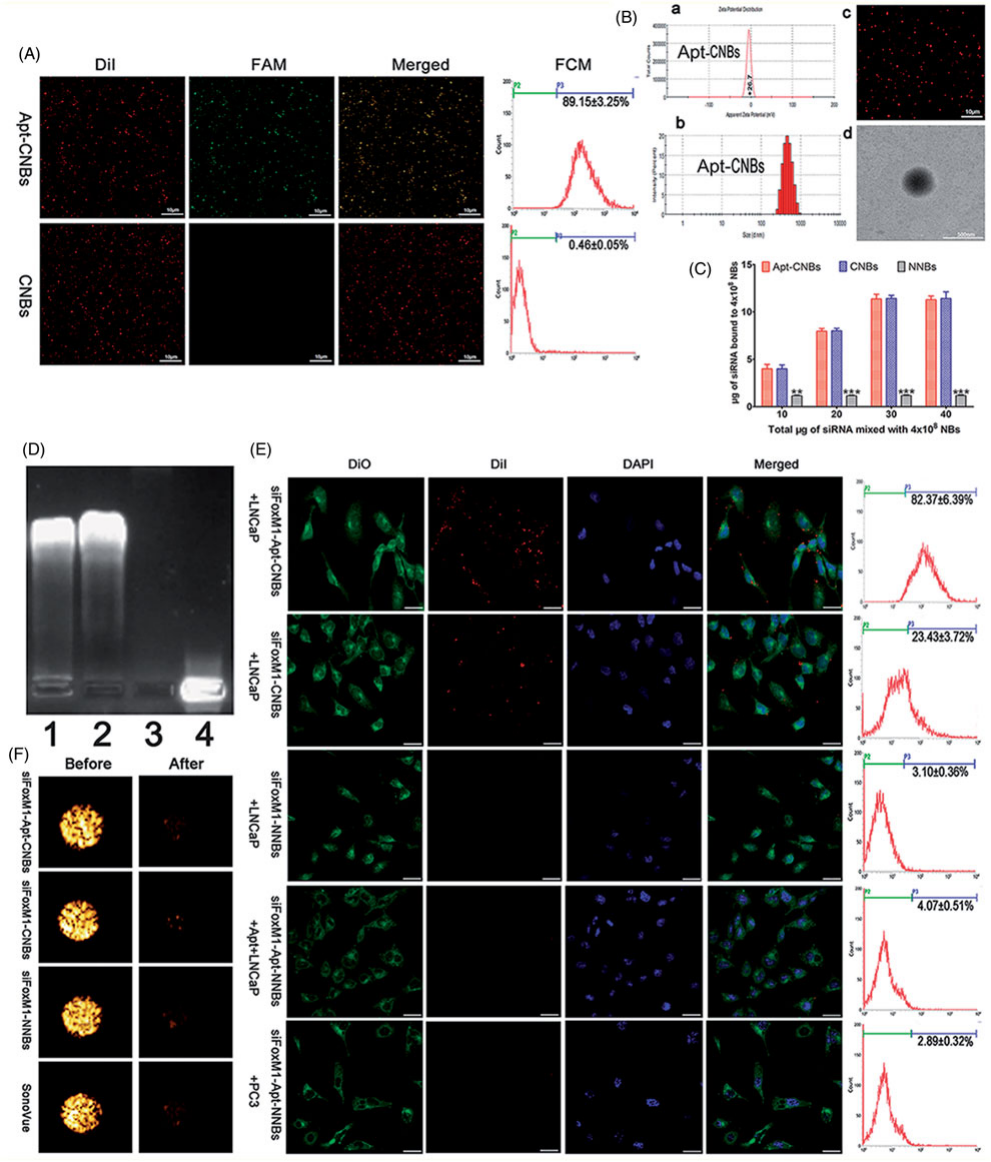
Synthesis of Apt-CNBs, characterization of Apt-CNBs, siFoxM1-loaded NBs, RNase protection, US sensitivity, target binding *in vitro*. (A) DiI-labeled carboxyl-modified CNBs (red), FAM-labeled amine-modified A10-3.2 aptamer (green) by covalently coupling reaction, overlapping the A10-3.2 aptamer using the CNBs emerging as yellow fluorescence in the mixed channel. (B) (a, b) Zeta potential and size distribution of the Apt-CNBs was determined by DLC. (c, d) LCSM and TEM image of DiI-labeled-Apt-CNBs. (C) The dose-dependent relationship between the FoxM1 siRNA and NBs. The loading capacity of the Apt-CNBs and CNBs with FoxM1 siRNA were significantly higher than the NNBs, **p < .01, ***p < .001, compared with NNBs. (D) FoxM1 siRNA via agarose gel electrophoresis. (1) siFoxM1-Apt-CNBs RNase (+) group, (2) siFoxM1-Apt-CNBs RNase (–) group, (3) naked FoxM1 siRNA RNase (+) group, (4) naked FoxM1 siRNA RNase (–) group. (E) Fluorescence imaging, together with the FCM, was put to use for evaluating the targeting efficacy of varied developments of siFoxM1-NBs to PSMA-positive LNCaP cells as well as PSMA-negative PC3 cells. Blue (DAPI) is a representation of cell nuclei, green (DiO) reveals the cytomembrane, whereas the red (DiI) dots imply DiI-labeled siFoxM1-NBs. (F) The US sensitivity of siFoxM1-Apt-CNBs did not change compared with that of siFoxM1-CNBs, siFoxM1-NNBs and SonoVue before and after low-frequency US destruction. *n* = 3; Scale bars =500 nm, 10 μm, or 20 μm.

LCSM revealed DiI-labeled CNBs (red color), in addition to the FAM-labeled A10-3.2 aptamer (green color). In respect of the mixed channel, the overlap of red as well as green is capable of appearing as yellow fluorescence (Figure 1(A)). Additionally, the efficiency with which the A10-3.2 aptamer bound to CNBs was 89.15%±3.25%, as recorded using FCM (Figure 1(A)).

### Characterization of the Apt-CNBs

Apt-CNBs were prepared using a thin-film hydration sonication and carbodiimide chemistry approaches method. The size distribution, zeta potential, PDI, and concentration zeta potential of the Apt-CNBs, CNBs, and NNBs were measured, and the results are listed in supplementary Table S2.

The average size of the Apt-CNBs was 479.83 ± 24.50 nm, with a PDI of 0.178 ± 0.023 (*n* = 3) (Figure 1(B(b)), Table S2). According to the DLS assessment, the low PDI indicated a high homogeneity in the size distribution of the Apt-CNBs. The average concentration of the Apt-CNBs was 4.05 ± 0.22 (10^8^/mL) (*n* = 3) (Table S2). No significant differences were found in the average diameter, PDI, or concentration between the Apt-CNBs, CNBs, and NNBs (*p* > .05) (Table S2). However, the zeta potential of the Apt-CNBs (24.07 ± 4.55 mV) was significantly higher than that of the NNBs (–4.45 ± 0.42 mV) (Figure 1(B(a)); Table S2) (*p* < .01). Furthermore, the higher zeta potential of the Apt-CNBs could combine with the negative charges of the siRNA. As the Apt-CNBs shell was labeled using a lipophilic red dye (Dil) to form DiI-labeled-Apt-CNBs, they could be more easily observed by LCSM, which showed that the distribution was uniform and that there was no obvious aggregation (Figure 1(B(c))). TEM showed that the Apt-CNBs were spherical (Figure 1(B(d))).

### FoxM1siRNA-binding ability of the Apt-CNBs

The siFoxM1-Apt-CNB complexes were developed with the use of an electrostatic adsorption strategy based on the negative charge of the siRNA and positive charge of the Apt-CNBs. The concentration of the Apt-CNBs was fixed (4 × 10^8^/mL) and the amount of FoxM1 siRNA was incrementally increased. With the addition of FoxM1 siRNA, the gene loading capacity of the Apt-CNBs increased gradually. When 30 μg of FoxM1 siRNA was added, the Apt-CNBs gene loading capacity no longer increased and approached saturation (Figure 1(C)). The loading capacity of the Apt-CNBs with FoxM1 siRNA was 11.28 ± 0.40 μg/4 × 10^8^. For the CNBs, NNBs, the above procedure was repeated, and the loading capacities of the CNBs and NNBs with FoxM1 siRNA were 11.42 ± 0.70 μg/4 × 10^8^ and 2.86 ± 0.12 μg/4 × 10^8^, respectively.

### RNase protection experiment

In the agarose gel electrophoresis analysis, the siFoxM1-Apt-CNBs RNase (+) and siFoxM1-Apt-CNBs RNase (–) groups showed evident bands, indicating that the Apt-CNBs protected the FoxM1 siRNA (Figure 1(D)). The naked FoxM1 siRNA RNase (+) group showed no obvious band, whereas the naked FoxM1 siRNA RNase (–) group showed a visible band (Figure 1(D)), indicating the RNase-mediated degradation of the exposed FoxM1 siRNA and confirming that free FoxM1 siRNA can be degraded by RNase in the absence of the Apt-CNBs vector. Thus, the Apt-CNBs protected the siRNA from being degraded by RNase and could function as suitable gene therapy vectors *in vivo*.

### *In vitro* assessment of target binding

Fluorescence imaging, together with the FCM, was put to use for evaluating the targeting efficacy of various types of siFoxM1-NBs with regard to PSMA-positive LNCaP cells, as well as PSMA-negative PC3 cells. As regards the group I, as revealed by the LCSM images, big quantities of DiI-labeled siFoxM1-Apt-CNBs (red fluorescent dots) exhibited their presence in the membranes (green fluorescence) that belong to LNCaP cells, which indicated that siFoxM1-Apt-CNBs are capable of targeting PSMA on the membranes of LNCaP cells, in addition to being later used by the cells (Figure 1(E)). In respect of group II, a small amount of red fluorescence DiI-labeled siFoxM1-CNBs was observed in the membranes of LNCaP cells shown in Figure 1(E). In group III, there were nearly no red DiI-labeled siFoxM1-NNBs on the LNCaP cells, which reflected the inability to target siFoxM1-NNBs to the cell membrane (Figure 1(E)). As regards the group IV, the inhibition/competitive group, siFoxM1-Apt-NNBs missed the potential of targeting the LNCaP cells as the PSMA was obstructed beforehand using extra free A10-3.2 aptamer, which threw light on the intracellular no DiI-labeled siFoxM1-Apt-NNBs (Figure 1(E)). Group V threw light on approximately no red DiI-labeled siFoxM1-Apt-NNBs present within PC3 cells, which reflected the shortage of PSMA expression by PC3 cells (Figure 1(E)). In addition, as revealed by FCM, the binding efficacies present between siFoxM1-NBs and the cells in groups I, II, III, IV, as well as V amounted to be 82.37% ± 6.39%, 23.43% ± 3.72%, 3.10% ± 0.36%, 4.07% ± 0.51%, and 2.89% ± 0.32%, respectively, shown in Figure 1(E). As brought forth by these *in vitro* observations, there is the direct proof of the exceptional targeting efficacy between siFoxM1-Apt-CNBs and PSMA-positive LNCaP cells.

### US sensitivity of the siFoxM1-Apt-CNBs

The *in vitro* gel tests showed that the siFoxM1-Apt-CNBs exhibited notably enhanced the contrast (Figure 1(F)). After 1 min of destruction to low-frequency US, the contrast intensity of the siFoxM1-Apt-CNBs, siFoxM1-CNBs, siFoxM1-NNBs, and SonoVue decreased significantly (Figure 1(F)). The intensity values of the siRNA-Apt-CNBs were not significantly different from those of the siFoxM1-CNBs, siFoxM1-NNBs, and SonoVue (*p* > .05) (Figures 1(F) and S1), indicating that low-frequency US can effectively destruct the siFoxM1-Apt-CNBs and that the siFoxM1-Apt-CNBs are very sensitive to low-frequency US (Figures 1(F) and S1).

### *In vitro* evaluation of transfection efficiency

**Figure 2.**
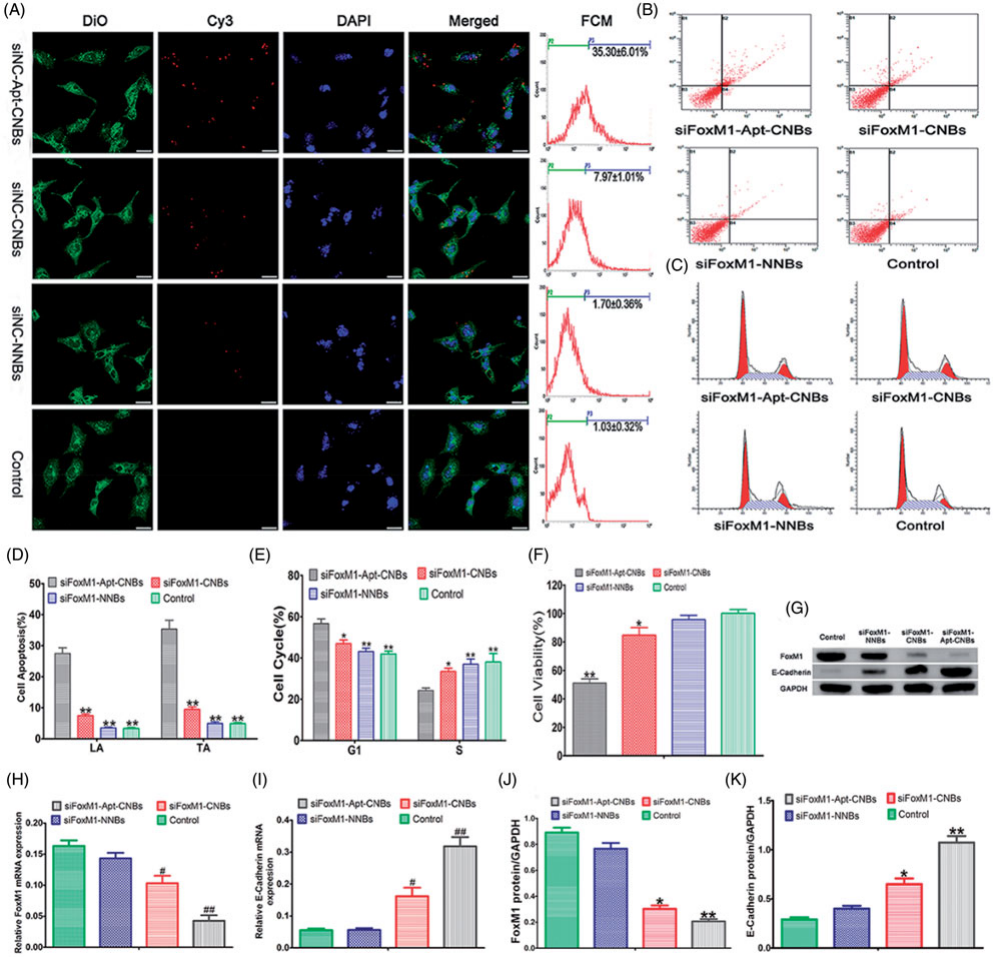
Transfection efficiency, cell apoptosis and cycle, cell viability, gene silencing efficiency of siFoxM1-Apt-CNBs *in vitro*. (A) Detection of the intracellular localization of Cy3-siRNA and measurement of the transfection efficiency through CLSM and flow cytometry, respectively. Blue (DAPI) is a representation of cell nuclei, green (DiO) reveals the cytomembrane, whereas the red (Cy3) dots imply Cy3-labeled FoxM1NCsiRNA. (B, C) The apoptotic and cycle of LNCaP cells were detected by flow cytometric after transfection for 48 h. (D, E) Quantitative flow cytometric analysis of the percentage of cell apoptotic and cell cycle changes after transfection for 48 h, *p < .05, **p < .01, compared with siFoxM1-Apt-CNBs groups, *n* = 3. (F) LNCaP cell viability was measured with CCK-8 after transfection treatment for 48 hours. (G, J, and K) The FoxM1, E-cadherin protein expression levels were detected by western blotting after 48 hours of transfection treatment. (H, I) The FoxM1 and E-cadherin mRNA expression levels were evaluated by RT-PCR after 48 hours of transfection treatment. ^#^*p* < .05, *p < .05 compared with the control, siFoxM1-NNBs and siFoxM1-Apt-CNBs groups; ##*p* < .01, **p < .01 compared with the control and siFoxM1-NNBs groups, *n* = 3; Scale bars =20 μm.

CLSM and FCM showed that compared with the control, a higher transfection efficiency was achieved in the siFoxM1-Apt-CNBs group. After transfection for six hours, red fluorescence was observed by CLSM, indicating that the Cy3-FoxM1 NC-siRNA transfection was successful. In the siNC-Apt-CNBs group, more red fluorescence was observed in the cytoplasm and nuclear periphery (Figure 2(A)), and a small amount of red fluorescence (Figure 2(A)) was observed in the siNC-CNBs group. No significant red fluorescence was observed in the siNC-NNBs and control groups (Figure 2(A)). After transfection of 24 hours, the quantitative flow cytometric analysis showed that the transfection efficiencies of the siNC-Apt-CNBs, siNC-CNBs, siNC-NNBs and control groups were 35.30 ± 6.01%, 7.97 ± 1.01%, 1.70 ± 0.36%, and 1.03 ± 0.32%, respectively. Therefore, the transfection efficiency of the siNC-Apt-CNBs group was 4.5 times higher as compared with that of the siNC-CNBs group in LNCaP cells (Figure 2(A)).

### Cell apoptosis and cycle analysis

As revealed by the quantitative flow cytometric analysis, in the LNCaP cells, the range of late apoptosis (LA) and total apoptosis (TA) showed an increasing trend: control < siFoxM1-NNBs < siFoxM1-CNBs < siFoxM1-Apt-CNBs (Figure 2(B,D)). The siFoxM1-Apt-CNBs group exhibited a substantially higher degrees of apoptosis than was seen in the FoxM1-CNBs group, indicating that A10-3.2 aptamer could further induce apoptosis (Figure 2(B,D)). In the PC3 cells, the LA and TA of the siFoxM1-Apt-CNBs were not significantly different from the siFoxM1-CNBs (Figure S2(A,C)); the LA and TA of the siFoxM1-Apt-CNBs and siFoxM1-CNBs were significantly compared with siFoxM1-NNBs and control (*p* < .05) (Figure S2(A,C)).

The cell cycle analysis showed that the siFoxM1-Apt-CNBs treatment of the LNCaP cells shifted a significantly high number of cells into the G1 phase, in addition to fewer cells into the S phase in comparison with that in the other groups (Figure 2(C,E)). As revealed by the quantitative flow cytometric analysis of the cell cycle, the allocation of the G1- and S-phase cells in the different groups after 48 hours was as follows: in the G1 phase, control < siFoxM1-NNBs < siFoxM1-CNBs < siFoxM1-Apt-CNBs; in the S phase, siFoxM1-Apt-CNBs < siFoxM1-CNBs < siFoxM1-NNBs < control (Figure 2(C,E)). The cells were grabbed in the G1 phase, proceeding with the decline in the quantity of cells in the S phase, which interfered with normal cell cycle proliferation and stimulated apoptosis. Therefore, siFoxM1-Apt-CNBs stimulate cell cycle arrest in the G1 phase, which becomes more obvious in cells treated with the targeted A10-3.2 aptamer. In the PC3 cells, in the G1 phase, the siFoxM1-Apt-CNBs were not significantly compared with that in the other groups (Figure S2(B,D)); in the S phase, the siFoxM1-Apt-CNBs and siFoxM1-CNBs were significantly compared with siFoxM1-NNBs and control (*p* < .05) (Figure S2(B,D)).

### Cell viability and cytotoxicity

CCK-8 was put to use for measuring the cell viability in response to transfection with FoxM1 siRNA. The siFoxM1-Apt-CNBs group showed lower cell viability (51.07 ± 4.97%) than the siFoxM1-CNBs (84.73 ± 9.12%), siFoxM1-NNBs (95.67 ± 5.12%) and control (100.17 ± 4.51%) groups (Figure 2(F)). The results indicated that siFoxM1-Apt-CNBs combined with US can inhibit LNCaP cell proliferation and promote apoptosis *in vitro*. However, CCK-8 cytotoxicity assay results revealed that siNC-Apt-CNBs, siNC-CNBs, siNC-NNBs, and siNC-Apt-CNBs + US, siNC-CNBs + US, siNC-NNBs + US had very low toxicity, the average viability of LNCaP cells was over 90% (Figure S3).

### RT-PCR and Western blotting analysis

The mRNA expression degrees of FoxM1 and E-cadherin were determined after a 48-hour transfection, as shown in Figure 2(H,I). The siFoxM1-Apt-CNBs group had a lower FoxM1 mRNA expression level than the siFoxM1-CNBs, siFoxM1-NNBs, and control groups (Figure 2(H)), further indicating that A10-3.2 aptamer during the transfection process plays a key role in reducing FoxM1 mRNA levels. Meanwhile, with the decrease in FoxM1 expression, the E-cadherin mRNA expression increased (Figure 2(I)). Thus, the expression of FoxM1 could inhibit the expression of E-cadherin.

Measurements of the protein expression degrees of FoxM1 as well as E-cadherin were carried out with the help of western blotting after a 48-hour transfection, as shown in Figure 2(G). As evident from Figure 2(G,J,K), the protein expression degrees of FoxM1 were lowest in the siFoxM1-Apt-CNBs group (Figure 2(G,J)), while the E-cadherin protein expression level was the highest (Figure 2(G,K)). Consistent with the PCR results, the analysis at the protein level showed that FoxM1 expression could inhibit E-cadherin expression.

### *In vivo* fluorescence imaging in the xenograft tumors

**Figure 3.**
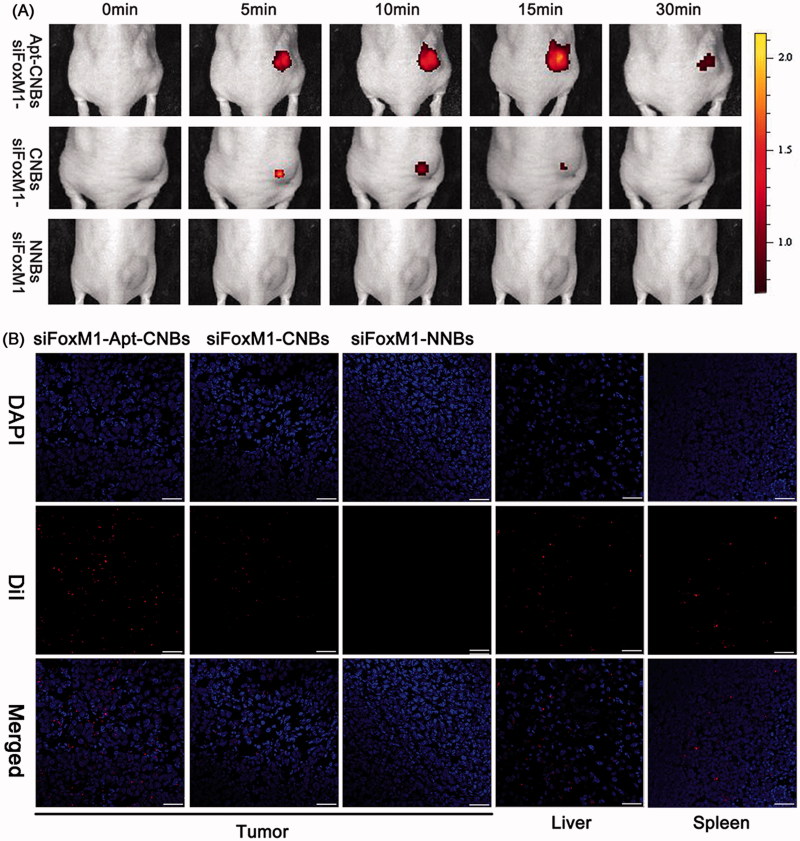
Fluorescence imaging and bio-distribution in the xenograft tumors (A) variations that happen in the fluorescence intensity of DiR-labeled siFoxM1-Apt-CNBs, siFoxM1-CNBs, and siFoxM1-NNBs in xenograft tumors in nude mice at the time intervals of 0, 5, 10, 15, and 30 min. (B) Biodistribution of DiI-labeled siFoxM1-Apt-CNBs, siFoxM1-CNBs, and siFoxM1-NNBs in the cryosections of varied organ tissues by LCSM. Blue (DAPI) is a representation of cell nuclei, whereas the red (DiI) dots imply DiI-labeled siFoxM1-NBs. Scale bars =20 μm.

As revealed by the tiny animal living fluorescence imaging, more DiR-labeled siFoxM1-Apt-CNBs accumulated in xenograft tumors and persisted for longer. However, less DiR-labeled siFoxM1-CNBs accumulated or did not endure and no DiR-labeled siFoxM1-NNBs accumulated in xenograft tumors, revealing lower fluorescence severity as compared with the siFoxM1-Apt-CNBs (Figure 3(A)). Moreover, DiR-labeled siFoxM1-NBs fluorescence intensity was observed in liver, spleen, kidney, organ tissues by the tiny animal living fluorescence imaging apparatus (Figure S4). LCSM demonstrated that more red DiI-labeled siFoxM1-Apt-CNBs accumulated in the tumor cryosections, whereas a smaller amount of DiI-labeled siFoxM1-CNBs and no red DiI-labeled siFoxM1-NNBs accumulated in the tumor cryosections (Figure 3(B)). Moreover, DiI-labeled siFoxM1-NBs were observed in liver and spleen tissue cryosections (Figure 3(B)). As revealed by these observations, the siFoxM1-Apt-CNBs are capable of doing specific detection of PSMA-expressing tumors, together with accumulation in tumor tissues, accordingly, thereby delivering more efficient treatment that is capable of inhibiting xenograft tumors.

### Therapeutic effect of FoxM1 siRNA *in vivo*

**Figure 4.**
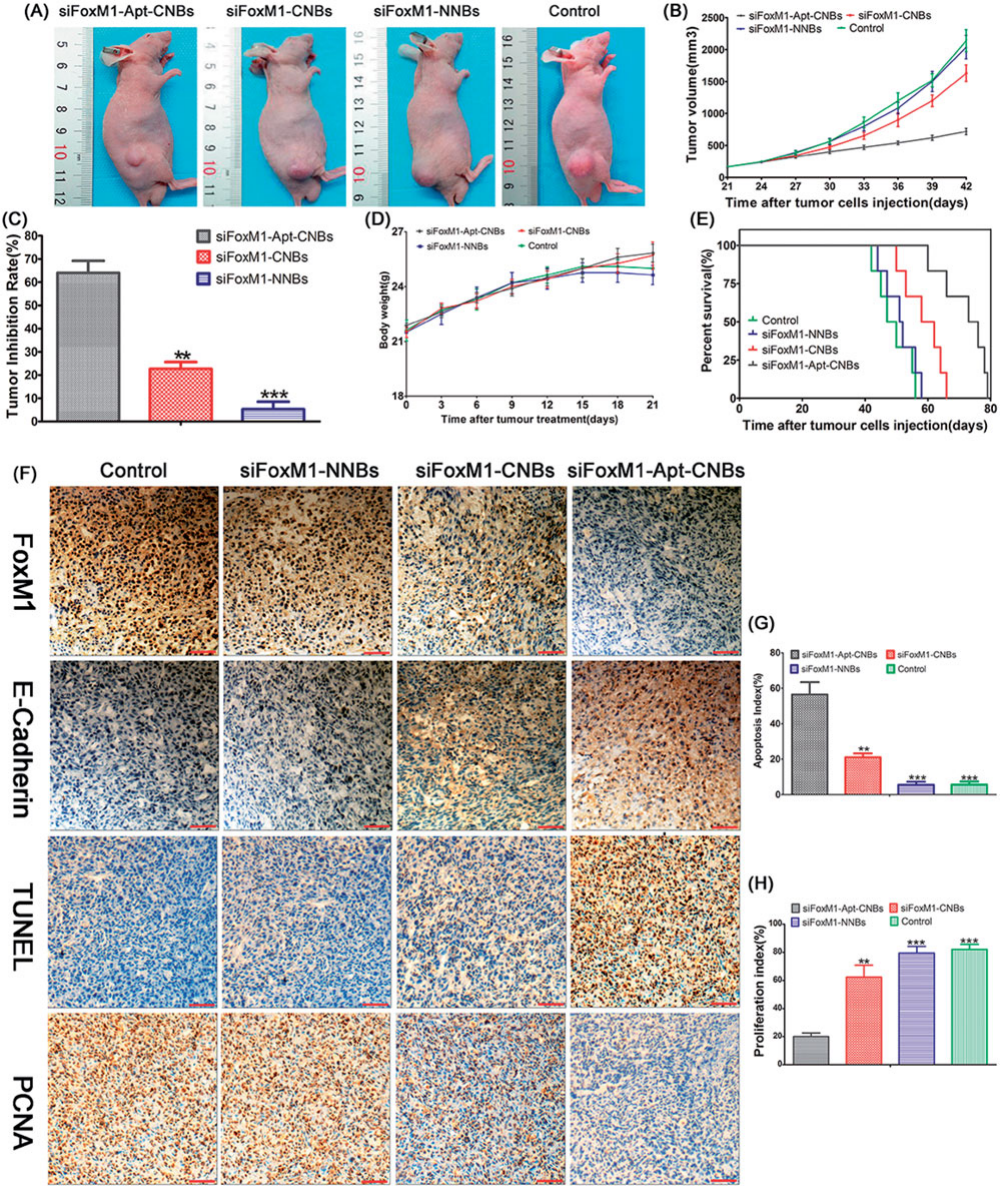
Therapeutic effect of siFoxM1-NBs combined with UMND *in vivo*. (A) Tumor volume in the various treatment groups. (B) Tumor growth curve in the various treatment groups. (C) Tumor inhibition rate in the various treatment groups. (D) Changes in the body weight in the various treatment groups. (E) Cumulative survival outcome of the LNCaP tumor-bearing nude mice in the various treatment groups. (F) Immunohistochemistry, TUNEL, and PCNA assay analyses of tumor tissue section from xenograft-bearing mice receiving different treatment after 21 days treatment. Analysis of immunohistochemical assays, brown staining indicates the positive expression of FoxM1, E-cadherin in tumor tissue, and the blue staining indicates the cell nuclei, respectively. Analysis of TUNEL and PCNA assays, brown staining the cell nuclei indicates the apoptosis- and proliferation-positive tumor cells, whereas blue staining the cell nuclei indicates the apoptosis- and proliferation-negative tumor cells. (G, H) Apoptosis and proliferation index of the various treatment groups, **p < .01, ***p < .001, compared with siFoxM1-Apt-CNBs group; scale bars =50 μm.

After repeating the treatments with siFoxM1-Apt-CNBs combined with UMND for gene therapy, tumor growth was effectively delayed (Figure 4(A)). After the repeated treatments in the different groups, tumor growth was observed, and growth curves were generated (Figure 4(B)). From day 6, the tumor growth rate in the siFoxM1-Apt-CNBs group amounted to be substantially slower in comparison with that existing in other cohorts, and the tumor volume amounted to be substantially smaller in comparison with that in the other groups (Figure 4(A,B)). After 21 days of treatment, the average tumor volume was 718.00 ± 114.54 mm^3^, 1630.00 ± 286.36 mm^3^ (*p* < .01), 2040.00 ± 414.40 mm^3^ (*p* < .001), and 2140.00 ± 384.71 mm^3^ (*p* < .001) in the siFoxM1-Apt-CNBs, siFoxM1-CNBs, siFoxM1-NNBs, and control groups, respectively. Moreover, the application of siFoxM1-CNBs with the A10-3.2 aptamer led to a TIR of 64.10%±11.47% (Figure 4(C)). The therapeutic effect in the siFoxM1-Apt-CNBs group was superior to that in the siFoxM1-CNBs (*p* < .01), siFoxM1-NNBs (*p* < .001), and control groups (Figure 4(C)), demonstrating that the A10-3.2 aptamer can improve the anti-tumor effect.

Blood biochemical tests and change of body weight measurements showed that no significant differences in ALB, ALT, CREA, AST, BUN, and body weight were found between the siFoxM1-Apt-CNBs group and control groups (Figures S5 and 4(D)), suggesting low toxicity of siFoxM1-Apt-CNBs.

Nude mice in the siFoxM1-Apt-CNBs, siFoxM1-CNBs, siFoxM1-NNBs, and control groups died on days 60, 50, 44, and 42, respectively. Compared with the other groups, the siFoxM1-Apt-CNBs group showed a better therapeutic effect; 50% of the mice survived for more than 73 days, and the longest survival time was 79 days (Figure 4(E)).

Thus, siFoxM1-Apt-CNBs combined with UMND gene therapy can improve both the transfection efficiency and the efficiency of gene delivery, which would meet the ultimate goal of tumor therapy.

Confirmations of the expression degrees of FoxM1 as well as E-cadherin in the nude mouse xenografts were carried out with the help of immunohistochemistry, with brown staining indicating the positive expression of FoxM1, E-cadherin in tumor tissue and blue staining indicating the tumor cell nuclei. The positive expression degrees of FoxM1 in the siFoxM1-Apt-CNBs group were lowered than in the other groups (Figure 4(F)). The E-cadherin positivity level in the siFoxM1-Apt-CNBs group was greater than the degrees in the other groups (Figure 4(F)). The number of apoptotic and proliferating cells in the nude mouse xenografts was confirmed by TUNEL and PCNA, respectively, with brown staining nuclei indicating the apoptosis- and proliferation-positive tumor cells and blue staining nuclei indicating apoptosis- and proliferation-negative tumor cells. The number of apoptosis-positive cells and the AI in the siFoxM1-Apt-CNBs group was higher than those in the other groups (Figure 4(F,G)) (*p* < .01, *p* < .001, compared with the siFoxM1-Apt-CNBs group). The number of proliferation-positive cells and the PI in the siFoxM1-Apt-CNBs group amounted to be quite lower in comparison with that in the other groups (Figure 4(F,H)) (*p* < .01, *p* < .001, compared with siFoxM1-Apt-CNBs group). The immunohistochemistry, TUNEL and PCNA results showed that the siFoxM1-Apt-CNBs combined with UMND significantly inhibited the expression of FoxM1, PCNA and effectively promoted E-cadherin and TUNEL positivity.

## Discussion

The combination of neutral microbubbles (NMBs) with UMMD, and the gene transfection efficacy appeared to be diffident, sharing similarity with what reported the other scholars (Bekeredjian et al., 2005). Nonetheless, owing to its benefits of noninvasive, non-viral as well as targeted transfection, continuous investigation of UMMD has been performed right from the time of its development. The benefits of non-viral transfection are capable of making its use in medical treatment a reality if its modest transfection efficiency can be improved. Accordingly, improvement in the transfection efficacy is emerging as a major point focus of prospective studies. Currently, NMBs and therapeutic genes are primarily combined through electrostatic adsorption, and because the current commonly used NMBs surface has a negative or no charge, the capacity for loading DNA, RNA, plasmids and other negatively charged particles is very low, which severely limits the application of NMBs as gene vectors (Yin et al., 2014).

Additionally, the micro-scaled NMBs impedes their passage through the endothelial gap into tumor tissue and the subsequent targeted release of therapeutic genes (Rapoport et al., 2007); thus, the transfer of drugs or genes into tumor cells to promote tumor treatment is difficult (Yin et al., 2013). Compared with NMBs, NNBs are smaller in size and can more easily penetrate the endothelial gap to reach tumor cells. Therefore, the construction of a highly US-sensitive and positively charged nanoscaled gene vector (Chumakova et al., 2008; Qiu et al., 2010) would not only improve the stability of the gene being carried but also increase the capacity for loading the gene.

Contrasting to other reports, we made a selection of DC-Chol for making the facade positively charged. This lipid is termed as among the most effective and commonly applied cationic lipids, together with having been extensively utilized in gene transfection as well as drug delivery (Caracciolo et al., 2010).

To overcome these challenges described above, we successfully prepared positively charged CNBs by adding positively charged DC-cholesterol to the phospholipid material on the film.

In terms of our process, an amount of 0.5 mg of DC-Chol was inserted for the construction of the CNBs, whereby the mean zeta-potential amounted to be 26.07 ± 4.21 mV. The nanobubbles demonstrated stability in suspension for a period of 1 week at a temperature of 4 °C. Subsequent to the increase in the quantity of DC-Chol, the zeta-potential of the CNB also showed growth. However, the stability demonstrated a decline. The zeta-potentials of CNBs were similar with the reports presented by other scholars (Zhou et al., 2015). There have been no former researches reporting that the stability of nanobubbles received impact from the potential. As per our speculation, this finding was likely to be owing to the fact that DC-Chol possesses more rigidity as compared with other cationic substances that was utilized by the other scholars for the construction of CNBs. Here, the siRNA loading potential of CNBs was more comparable to that of NNBs. Nonetheless, we also presented the report that the mix of siRNA approached the saturation with the addition of 30 µg siRNA. The zeta-potentials reports made by all of the scholars had differences (Christiansen et al., 2003; Nomikou et al., 2012; Panje et al., 2012; Xie et al., 2012), in addition to the siRNA loading potentials as well as transfection efficacies. Accordingly, the association between the zeta-potential, siRNA loading potential and transfection efficacy of CNBs needs additional research as well. In the agarose gel electrophoresis experiment, the Apt-CNBs protected the siRNA from being degraded by RNase. The high siRNA loading capacities protected the siRNA, which indicated that CNBs are a good gene vector in UMND gene therapy for *in vivo* tumor.

One noteworthy characteristic associated with our CNBs suggests that it was developed with DSPE-PEG2000-COOH instead of DSPE-PEG2000, together with having a carboxyl group of the cationic shell. We made a selection of DSPE-PEG2000-COOH rather than DSPE-PEG2000 for the construction of CNBs not just owing to the fact that the addition of the carboxyl group tag allowed molecular targeting strategies but also due to the reason that the density of CNBs amounted to be higher, together with improved stability. Owing to the reason that biotin–avidin is termed as an exogenous protein for the induction of immunological reactions as we utilized, the development of immune complexes in the lowest membrane of kidney is quite convenient that constrains the consumption of biotin–avidin in human body. Accordingly, we made a choice of amine-modified A10-3.2 aptamer molecules in our practical approach because of the extreme specificity, less immunogenicity, as well as stability of the reaction between the amine and carboxyl groups, which ensures the link between the CNBs and the aptamers. The combination of the A10-3.2 aptamer with the CNBs exhibited no impact on the siRNA binding potential of CNBs (Figure 1(C)). Additionally, no impact was received by the targeting potential of Apt-CNBs to LNCaP cells from the mix of siRNA (Figure 1(E)). The phenomena targeting LNCaP differed between siRNA-Apt-CNBs and siRNA-CNBs. In *in vitro* evaluation of the target binding practical approach, we noticed nearly no siRNA-NNBs within LNCaP cells. However, a quantity of siRNA-CNBs was linked to the LNCaP cells; the dissimilarity between the siRNA-NNBs and siRNA-CNBs groups appeared to be considerable. A large amount of targeted siFoxM1-Apt-CNBs attached to the LNCaP cells, and the number of attached siFoxM1-Apt-CNBs was 3.7-fold higher than that for siRNA-CNBs, the difference between the siFoxM1-Apt-CNBs and siFoxM1-CNBs groups was significant. These results revealed that siFoxM1-CNBs could target to LNCaP cells through electrostatic interaction (Nomikou et al., 2012); the primary mechanism through which siFoxM1-Apt-CNBs targeting to LNCaP cells was the reaction between the aptamer and antigen; we hold the belief that the reaction between the antigen and aptamer is considered as the key phenomenon as well as the actual target reaction, however, the mechanism through which Apt-CNBs targeting to LNCaP cells may still include electrostatic interactions; the *in vivo* small animals live fluorescence imaging, together with LCSM additionally validated the targeted selectivity of the siFoxM1-Apt-CNBs for PSMA-positive tumors.

In terms of the cell viability test, we noticed the fact that the LNCaP cells viability exhibited a decrease following the treatment with siFoxM1-Apt-CNBs and was considerably lower than that observed following treatment with the siRNA-CNBs, validating the fact that augmenting the number of target-attached nanobubbles is capable of decreasing the cell viability, which has been previous indicated in reports (Li et al., 2009; Tlaxca et al., 2010; Nomikou et al., 2012). Transfection efficiency exhibited an increase with the decrease in cell viability. Analyzing the impact of these therapeutic tools *in vitro* demonstrated the exceptional therapeutic potential of siFoxM1-Apt-CNBs. Despite the fact that the therapeutic impact using with siFoxM1-Apt-CNBs appeared to be better as compared with the impact that used siFoxM1-CNBs *in vitro*, yet the benefit of siFoxM1-Apt-CNBs while used *in vivo* has more importance. Despite the fact that siFoxM1-CNBs are capable of targeting LNCaP cells *in vitro* with the help of electrostatic interaction, this benefit no longer appeared *in vivo*. Moreover, owing to the boosted phagocytosis by reticuloendothelial system, the siFoxM1-CNBs would be immediately metabolized. The above-stated constraints associated with siFoxM1-CNBs were combined with UMND gene therapy *in vivo*. As the siFoxM1-CNBs were not capable of targeting cells in the therapeutic application, the use of siFoxM1-Apt-CNBs was of much greater benefit *in vivo* than was apparent *in vitro*. The inhibition rate with siFoxM1-Apt-CNBs was approximately three-fold that of siFoxM1-CNBs, together with the observation of an apparent inhibition of tumor proliferation (Figure 4(C)).

To verify the impact of the siFoxM1-Apt-CNBs with regard to cytotoxicity *in vitro* and systemic toxicity *in vivo*, we performed the evaluation of siNC-NBs or siNC-NBs + US incubated with LNCaP cells for 48 hours, and the results showed very low toxicity (Figure S3). We also performed the evaluation of the nude mice weight daily and serum blood biochemical tests after the siFoxM1-Apt-CNBs were combined with UMND gene therapy *in vivo*. The changes of weight and blood biochemical tests indicated that no significant difference in weight, ALB, ALT, AST, BUN, and CREA was found between the experimental groups and the control groups (Figures 4(D) and S5), suggesting low toxicity of siFoxM1-Apt-CNBs, without serious side effects.

The dissimilarities in the therapeutic impact between siFoxM1-CNBs and siFoxM1-Apt-CNBs *in vivo* amounted to be more substantial in comparison with that *in vitro*. After treatment with Apt-CNBs loaded with FoxM1siRNA, the observed increase in transfection efficiency greatly increased the E-cadherin expression level and positive TUNEL staining, while decreasing the FoxM1 expression levels and positive PCNA staining. Therefore, FoxM1 gene therapy with the use of UMND is likely to result in an effective methodology for anti-proliferation therapy.

Thus, siFoxM1-Apt-CNBs combined with UMND achieved a good therapeutic effect in LNCaP cells and xenografts tumor by silencing the expression of FoxM1.

## Conclusions

In conclusion, we successfully prepared uniform nanoscaled siFoxM1-Apt-CNBs, which have a high affinity and specificity for the PSMA-positive LNCap cells and xenografts tumors in nude mice *in vitro* and *in vivo*. We have demonstrated that siFoxM1-Apt-CNBs combined with UMND can effectively decrease the expression of FoxM1 and increase in E-cadherin expression *in vitro* and *in vivo*. In addition, siFoxM1-Apt-CNBs combined with UMND successfully inhibited cell proliferation and promoted apoptosis *in vitro*, while tumor growth obstruction and low toxicity were also taken into observation *in vivo*. Collectively, siFoxM1-Apt-CNBs combined with UMND represent a novel tumor-targeting delivery system for siRNAs and are therefore a promising tool for cancer gene therapy.

## Supplementary Material

IDRD_Tang_et_al_Supplemental_Content.doc
