# Supplementary material for: Ultrasound-mediated nanobubble destruction (UMND) facilitates the delivery of A10-3.2 aptamer targeted and siRNA-loaded cationic nanobubbles for therapy of prostate cancer
Source: Drug Deliv. 2018 Jan 9;25(1):226–40. doi: 10.1080/10717544.2017.1422300 (PMC6058493; doi:10.1080/10717544.2017.1422300)
Supplement: IDRD_Tang_et_al_Supplemental_Content.doc [file IDRD_A_1422300_SM6579.doc]

**Table S1.** The primer sequences of FoxM1, E-Cadherin detected by

| | Primer Sequence | | --- | | FoxM1 F GCAGCGACAGGTTAAGGTTGAG  FoxM1 R GAAAGGTTGTGGCGGATGGAGT  E-Cadherin F CCAATCCCGATGAAAT  E-Cadherin R CATAGTCAAACACGAGCA  GAPDH F CCACTCCTCCACCTTTG  GAPDH R CACCACCCTGTTGCTGT | |
| --- | --- | --- |

**Table S2.** Characterization of the nanobubbles

| Group Size distribution (nm) Zeta potential (mV) PDI Concentration (10^8^/mL) |
| --- |
| Apt-CNBs 479.83±24.50 + 24.07±4.55** 0.178±0.023 4.05±0.22  CNBs 463.25±30.24 26.54±4.21** 0.185±0.022 4.10±0.18  NNBs 483.37±20.11 -4.45±0.42 0.193±0.015 3.95±0.13 |

Notes: **P<0.01, compared with the NNBs.


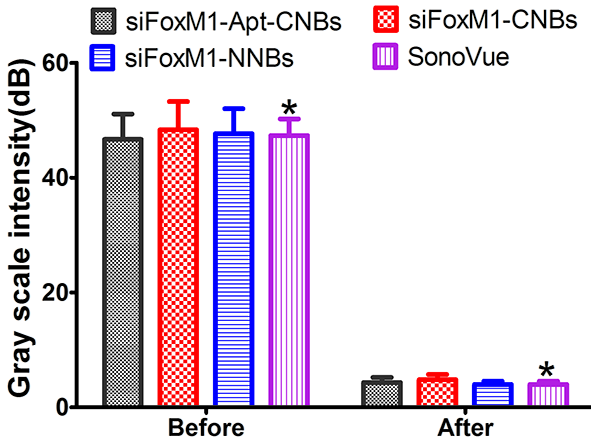


**Figure S1.** Quantitative analysis of the greyscale intensity values among the siFoxM1-Apt-CNBs, siFoxM1-CNBs, siFoxM1-NNB and SonoVue groups before and after low-frequency US destruction in vitro (*P>0.05, compared with SonoVue group).


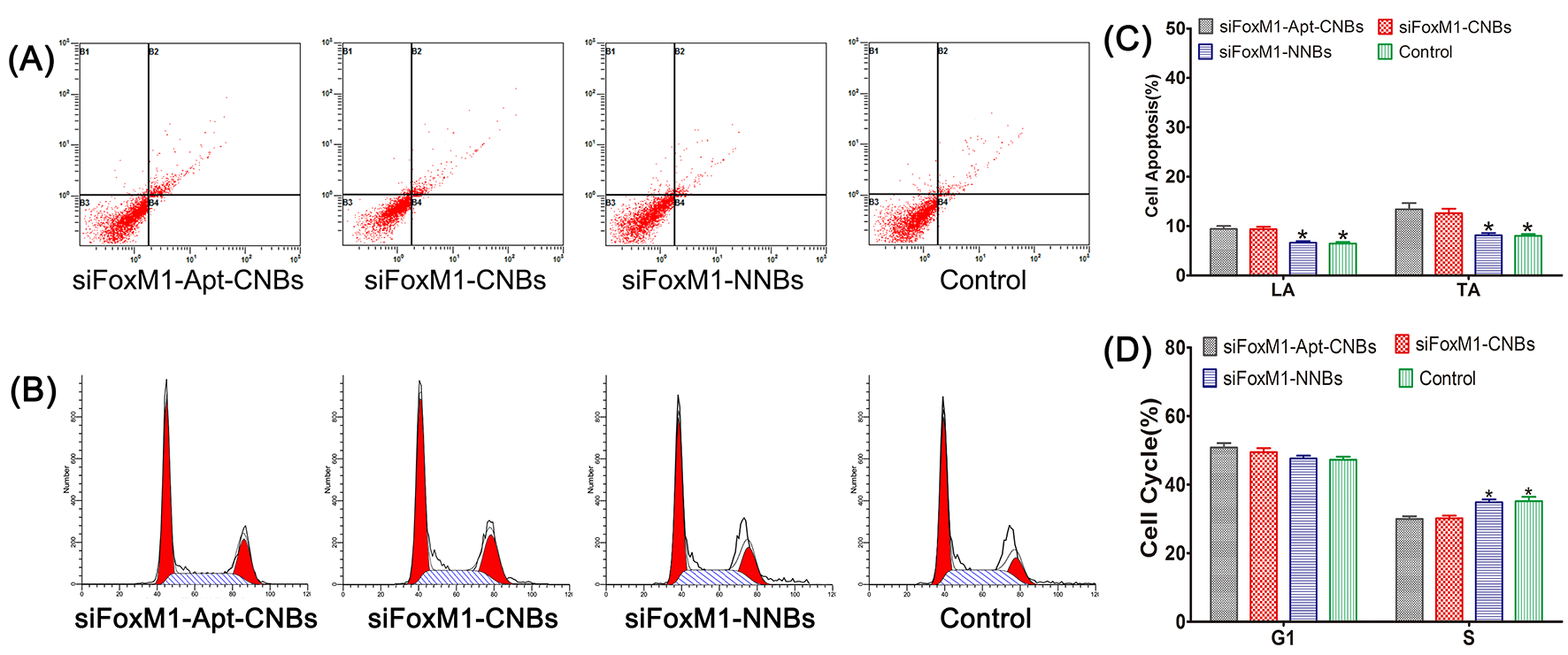


**Figure S2.** Quantitative flow cytometric analysis of the percentage of cell apoptotic and cell cycle changes after transfection for 48 h for PC3 cells. (A-B) The apoptotic and cycle of PC3 cells were detected by flow cytometric after transfection for 48 h. (C-D) Quantitative flow cytometric analysis of the percentage of cell apoptotic and cell cycle changes after transfection for 48 h, *P < 0.05, compared with siFoxM1-Apt-CNBs and siFoxM1-CNBs groups, n=3.

**
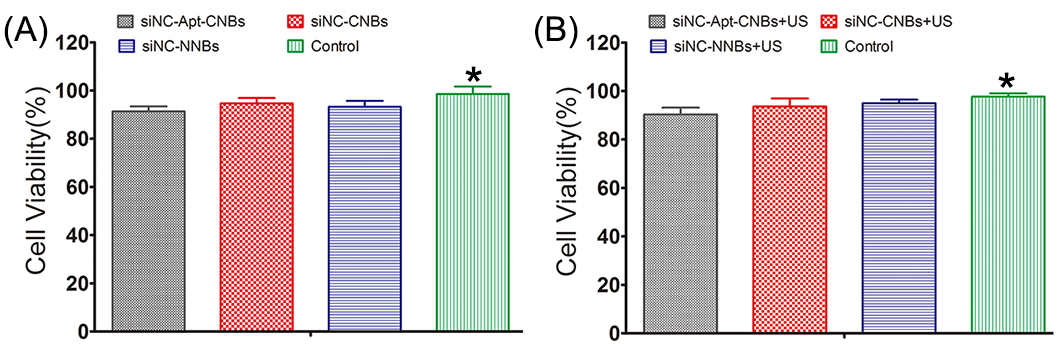
**

**Figure S3.** Cytotoxicity in vitro. LNCaP cells were incubated with siNC-Apt-CNBs, siNC-CNBs, and siNC-NNBs (A), siNC-Apt-CNBs+US, siNC-CNBs+US, and siNC-NNBs+US (B) For 48 hours, and cell viability was analyzed using a CCK-8 assay. (*P > 0.05, compared with control group)


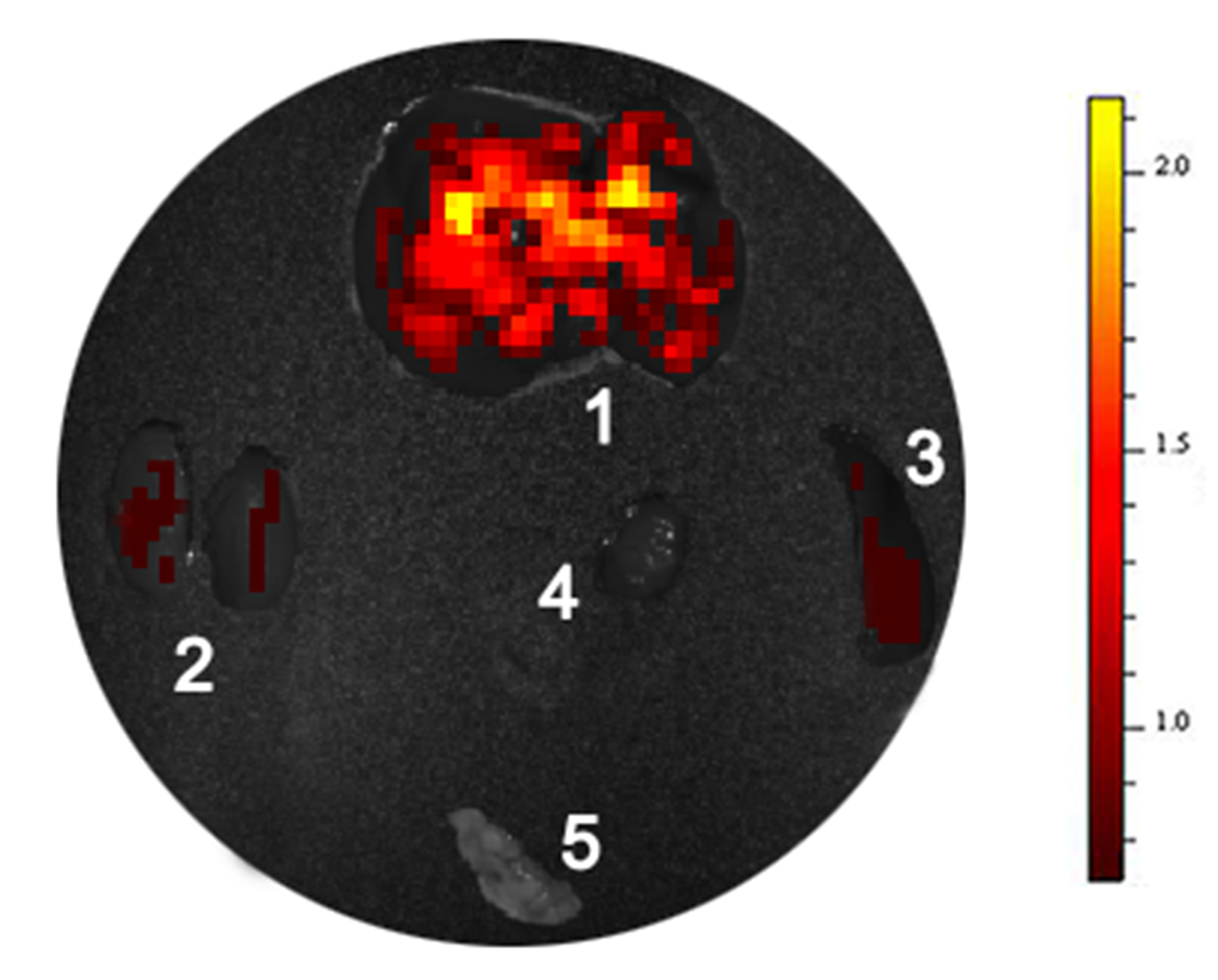


**Figure S4.** Fluorescence imaging intensity of DiR-labeled siFoxM1-NBs in the liver, spleen, kidney, heart, lung organ tissues by the tiny animal live fluorescence imaging apparatus. 1. liver; 2. kidneys; 3. spleen; 4. heart; 5. lung.


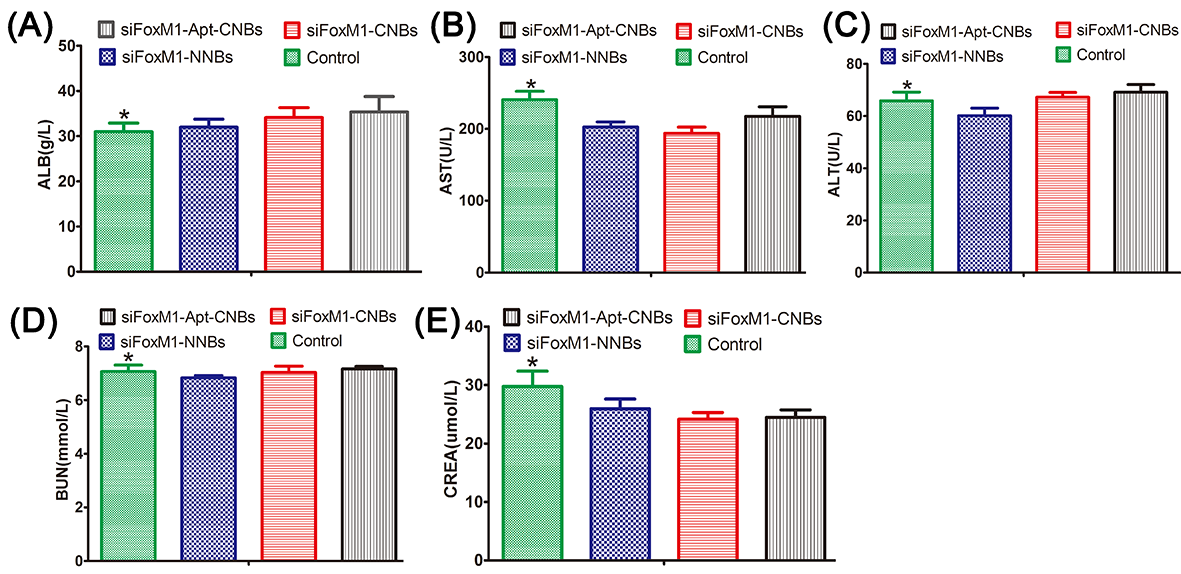


**Figure S5.** Serum markers indicated the functions of liver and kidney after the siFoxM1-Apt-CNBs combined with UMND gene therapy for 7 times in vivo. (A-E) ALB, ALT, AST, BUN, CREA levels in serum have no significant difference compared with the control group.(*P > 0.05, compared with control group)
